# Prevalence of Priapism and Its Awareness amongst Male Homozygous Sickle Cell Patients in Lagos, Nigeria

**DOI:** 10.1155/2013/890328

**Published:** 2013-07-15

**Authors:** Adewumi Adediran, Kikelomo Wright, Akinsegun Akinbami, Adedoyin Dosunmu, Olajumoke Oshinaike, Bodunrin Osikomaiya, Sarah Ajibola, Kamal Ismail, Ebele Uche, Olaitan Ojelabi

**Affiliations:** ^1^Department of Haematology and Blood Transfusion, Faculty of Clinical Sciences, College of Medicine, University of Lagos, PMB 12003, Surulere, Idi Araba, Lagos, Nigeria; ^2^Department of Community Medicine and Primary Health Care, Lagos State University College of Medicine, Ikeja, Nigeria; ^3^Department of Haematology and Blood Transfusion, Lagos State University College of Medicine, Ikeja, Nigeria; ^4^Department of Medicine, Lagos State University College of Medicine, Ikeja, Nigeria; ^5^Department of Haematology and Blood Transfusion, Lagos University Teaching Hospital, Ikeja, Nigeria; ^6^Department of Haematology and Blood Transfusion, Lagos University Teaching Hospital, Idi Araba, Nigeria

## Abstract

*Background*. Priapism is a pathological condition of penile erection that persists beyond, or is unrelated to, sexual stimulation. Impotence and infertility are major problems in male sickle cell disease patients, and priapism has been implicated as a cause of impotence and infertility. The aim of this study is to determine priapism prevalence and assess the knowledge of male homozygous male patients about it in Lagos, Nigeria. *Methods*. A cross-sectional study was conducted amongst male homozygous sickle cell disease patients of Lagos State University Teaching Hospital. Pretested questionnaires were distributed to determine the prevalence and assess their knowledge on priapism. Data were analyzed using SPSS version 16.0. *Results*. A total of 114 consenting subjects filled the questionnaires, 85 of 114 (74.6%) had not heard about priapism before this study. A total of 77 of 114 (67.5%) did not know that they are at risk of priapism. Whilst 84 of 114 (73.7%) were not aware that priapism is a complication of SCD. The majority, 94 of 114 (82.5%), were not aware that priapism could cause impotence. *Conclusion*. There is a need to create more awareness about this complication amongst sickle cell disease patients in order to stem the incidence of impotence and infertility amongst them.

## 1. Background

### 1.1. Sickle Cell Disease

Sickle cell disorder is a genetic abnormality involving haemoglobin and red cells. The glutamine on the 6th position of the *β* chain is replaced with valine consequent on a single point mutation in which thymine replaces adenine on the deoxyribonucleic acid structure. Haemoglobin S resulting from the substitution causes polymerization of haemoglobin and red cell sickling on exposure to low-oxygen tension and unsickle on oxygenation.

The sickled red cells obstruct blood vessels and impede free flow of blood of the affected vessel causing vascular congestion, oxygen deprivation, anaerobic glycolysis, lactic acidosis, and pain. This may be responsible for priapism, an acute complication of sickle cell disease.

### 1.2. Priapism in Sickle Cell Disease

Priapism is a pathological condition of penile erection that persists beyond, or is unrelated to, sexual stimulation [1]. Two subtypes have been described, the high flow (nonischemic) and the low flow (ischemic). The latter is associated with sickle cell disease and patients are more susceptible to greater complications and the long term recovery is dependent on prompt and urgent intervention [1]. A high prevalence of 42% rising to 50% was reported amongst Jamaican patients [2]. Its incidence varies between 5–45% [3]. A bimodal age distribution of 5–13 years and 21–29 years was also noted amongst Jamaicans [2].

Priapism is due to occlusion of the outflow vessels from the corpora cavernosa by sickled cells, consistent with significantly lower HbF levels in affected cases [2]. The resulting static reservoir becomes deoxygenated, and blood aspirated from the corpora cavernosa during priapism yields a thick dark-red viscous fluid, generally not clotted but consisting of irreversibly sickled cells [4].

Priapism may be self limited, and of short duration usually lasting less than 3 hours. It may also be chronic and recurrent lasting more than 24 hours. Increased rate of impotence has been reported in sickle cell patients whose attacks lasted more than 24 hours [5]. The prevalence of impotence in sickle cell disease is more than 25% [6]. When the glans and corpus spongiosum are spared from engorgement affecting only the paired cavernous bodies, it is referred to as bicorporal priapism. However, when engorgement affects corpus spongiosum, it is associated with poor prognosis and referred to as tricorporal priapism [7]. Recurrent trapping of irreversible sickled cells in the corpora cavernosa may lead to fibrosis of the septa and impotence [8].

Sexual intercourse has been implicated to precipitate attack amongst Jamaican. Others reported spontaneous attacks that wake the patient from sleep [2].

Many treatment modalities are available for priapism, these include the use of drugs, exchange blood transfusion and surgical intervention. Drugs like *α*-adrenergic agonist which increase the contractile state of the trabecular/arterial meshwork and facilitate venous outflow from the corpora [3] have been used. Other drugs tried with varying degree of successes are stilbesterol [9] the gonadotrophin-releasing hormone analogue leuprolide acetate [10] and anti-androgens [11].

Adequate hydration and analgesia may dislodge the trapped sickled red cells, partial exchange blood transfusion to lower the HbS level to less than 30% has been tried [12].

Surgical interventions which is usually the last procedure for unresolved priapism involves aspiration and irrigation of the corpora cavernosa with saline [13]. Creation of shunt or introducing a venous bypass in the cavernous spongiosum has been used to achieve cure [14]. Penile prosthesis is available after 6–12 months of impotence [15].

Infertility is a major problem in male sickle cell disease patients reported by Abudu et al. in which they noted a lower testosterone levels in males compared with age-matched controls [16]. Impotence has also been reported in about a quarter of sickle cell disease patients secondary to priapism [6]. This underscore the importance of priapism and the need to determine its prevalence amongst male sickle cell disease patients in Lagos and also assess their degree of knowledge about it, in order to enlighten them about the risk involved. Perhaps, they may seek early medical intervention in the event of priapism.

## 2. Methods

This cross-sectional study to determine the prevalence and assess the knowledge of male sickle cell disease patients on priapism was conducted amongst male homozygous sickle cell patients attending the sickle cell clinic of Lagos State University Teaching Hospital between February and November 2012. All participants were homozygous HbSS genotype. Consenting participants were recruited consecutively into the study after obtaining the institution's ethics and research committee approval. Pretested questionnaires were distributed to them to determine the prevalence and assess their knowledge on priapism. The questionnaire was designed by the authors, adapted from previous related studies [17–19], and adjusted to local setting. The questionnaires were pretested in a different community (urology clinic) from the study population by the authors for congruency and exclusion of ambiguities. It was refined thereafter and applied in the target population.

## 3. Statistical Assessment

Data were analyzed using SPSS version 16.0 (Statistical Package for Social Sciences, Inc., Chicago, IL, USA). The descriptive data was given as mean ± standard deviation (SD). The Pearson chi-squared test was used to test for association between discrete variables. A *P* value was considered to be statistically significant when <0.05.

## 4. Result

A total of 114 consenting subjects filled the questionnaires; the mean age was 21.97 ± 7.51, with a minimum of 14 years and maximum of 53 years. A total of 105 of 114 (92.1%) were single, while 9 of 114 (7.9%) were married. The majority, 65 of 114 (57%), had secondary education, 36 of 114 (31.6%) had tertiary education, 10 of 114 (8.8%) had primary education, while 3 of 114 (2.7%) had no education (Table 1).

A total of 85 of 114 (74.6%) of participants had not heard about priapism before this study, while only 26 of 114 (22.8%) had heard about it. Out of those who heard about it, about 11 of 114 (10%) heard from medical personnel and had personal experience, 4 of 114 heard from friends, and 1 of 114 (0.9%) heard from radio, textbook, and magazine.

The majority, 90 of 114 (78.9%), did not know what priapism is all about, while 24 of 114 (21%) knew what it is all about (Table 2). A total of 77 of 114 (67.5%) did not know that they are at risk of having priapism, while 36 of 114 (31.6%) knew, 1 of 114 (0.9%) did not answer the question. Out of 36 who knew that they were at risk of having priapism, 18 of 36 had secondary education, 16 of 36 had tertiary education, and 1 of 36 had primary education and no education *P* value 0.198. A substantial percentage of the participants 84 of 114 (73.7%) were not aware that priapism is a complication of SCD while 30 of 114 (26.3%) were aware. The majority (16/30) of the participants that were aware of priapism as a complication of SCD had secondary education, 13/30 had tertiary education, 1/30 had no formal education, while none of them had primary education; this was not statistically significant; *P* value was 0.17. A total of 19/30 Christians were aware of priapism, and 11/30 Muslims were aware; *P* value was 0.43. About 20/30 of participants who were aware of priapism were single and 5/30 married; this was statistically significant; *P* value was 0.038.

The majority, 94 of 114 (82.5%), were not aware that priapism could cause impotence, while 20 of 114 (17.6%) were aware that it could cause impotence. A total of 72 of 114 (63.2%) of participants were not sexually active while 42 of 114 (36.8%) were sexually active. Only 45 of 114 (39.1%) of participants had priapism in the past while 69 of 114 (60.5%) never had it (Table 2). Priapism resolved in less than 3 hours in 25 of 45 (55.5%), more than 3 hours but less than 24 hours in 7 of 45 (26.66%), and priapism lasted for more than 24 hours in 8 of 45 (17.7%). The mean age of first episode was 18.15 ± 5.03, the minimum age of first episode was 7 years, and the maximum was 30 years.

A total of 23 of 45 (51.11%) did not get any treatment, 6 of 45 (13.33%) were admitted, and 8 of 45 (17.77%) were treated but not admitted. When asked about how many times they had episode of priapism, the majority, 11 of 45 (24.44%), had it twice followed by 9 of 45 (20%) once, 4 of 45 (8.8%) thrice, and 3 of 45 (6.66%) 5 times. When asked about precipitating factors, 17 of 45 (37.77%) had it after waking up from sleep followed by no known factors in 10 of 45 (22.22%), followed by 5 of 45 (11.11%) following bone pain crises, and 4 of 45 (8.88%) after sexual intercourse.

## 5. Discussion

A prevalence of 39.1% reported in the current study is keeping with 35% reported by Adeyoju et al. in 2002 [17]. It is also similar to 42% reported amongst Jamaican patients [2] and much higher than 26.3% reported amongst Togolese in 2001 [18].

Knowledge about priapism was very poor amongst this study's participants; despite the fact that 88.6% had both secondary and tertiary education and could be considered literate, 74.6% of them had not heard about the word priapism before the current study, secondly, 78.9% of participants knew nothing about it, while 73.7% were unaware that it is a complication of sickle cell disease. This is similar to the study of Gbadoe et al. in 2007 who reported that only 10.2% of sickle cell patients knew about priapism and most were unaware of its association with sickle cell disease [19]. The implication of the poor knowledge about priapism is a late presentation associated with poor prognosis and a higher risk of impotence. All patients (100%) managed for priapism presented late at a tertiary health centre in Nigeria as reported by Badmus et al. in 2003 [20]. Similarly, Aghaji [21] reported on priapism amongst Nigerians that 21 of 35 patients presented to the hospital 6–10 days after the onset of erection because many did not realize that priapism was abnormal.

The mean age of the first episode reported in the current study was 18 ± 5.03 years which is similar to that of 15 years reported by Adeyoju et al. in 2002 [17]. However, it fell slightly outside the age range of 21–29 years reported amongst Jamaican [2]. Unlike the multicentre study [17], in which priapism was precipitated in most of the participants by sexual activity, it was precipitated in most of the participants in this study by waking up from sleep. Priapism after sexual activity constituted only 8.8%. This may be due to the fact that only 36.8% were sexually active in this study.

More than half of the participants had stuttering priapism because the current study reported that priapism resolved in less than 3 hours in more than half of the participants (55.5%), similarly, more than half of the participants who reported previous history of priapism (51.1%) had self-limiting priapism. Stuttering priapism is the recurrent, self-limiting episodes, which characteristically last for less than three hours, and is commonly seen in sickle cell disease [22].

## 6. Conclusion

Given the high prevalence of priapism amongst sickle cell patients in Lagos vis-à-vis their poor knowledge about it, there is a need to create more awareness about this complication amongst sickle cell disease patients in order to stem the incidence of impotence and infertility amongst them.

## Figures and Tables

**Table 1:** Sociodemographic characteristics of participants.

Parameters	
Marital status	
Single	105 (114), 92.1%
Married	9 (114), 7.9%
Age	
Minimum	14
Maximum	53
Mean	21.97 ± 7.51
Educational background	
Primary	10 (114), 8.8%
Secondary	65 (114), 57%
Tertiary	36 (114), 31.6%
None	3 (114), 2.7%

**Table 2:** Assessment of knowledge/prevalence about priapism.

	Yes	No
Knowledge about priapism	24 (114), 21%	90 (114), 78.9%
Knowledge of risk of priapism	36 (114), 31.6%	77 (114), 67.5%
Had priapism	45 (114), 39.1%	69 (114), 60.5%
Age at 1st episode		
Minimum	7	
Maximum	30	
Mean	18.15 ± 5.03	
